# Software Requirements Classification Using Machine Learning Algorithms

**DOI:** 10.3390/e22091057

**Published:** 2020-09-21

**Authors:** Edna Dias Canedo, Bruno Cordeiro Mendes

**Affiliations:** Department of Computer Science, University of Brasília (UnB), P.O. Box 4466, Brasília 70910-900, Brazil; brunocordeiro180@gmail.com

**Keywords:** functional requirements, non-functional requirements, text normalization, feature extraction, machine learning, support vector machines

## Abstract

The correct classification of requirements has become an essential task within software engineering. This study shows a comparison among the text feature extraction techniques, and machine learning algorithms to the problem of requirements engineer classification to answer the two major questions “Which works best (Bag of Words (BoW) vs. Term Frequency–Inverse Document Frequency (TF-IDF) vs. Chi Squared (${\mathrm{CHI}}^{2}$)) for classifying Software Requirements into Functional Requirements (FR) and Non-Functional Requirements (NF), and the sub-classes of Non-Functional Requirements?” and “Which Machine Learning Algorithm provides the best performance for the requirements classification task?”. The data used to perform the research was the PROMISE_exp, a recently made dataset that expands the already known PROMISE repository, a repository that contains labeled software requirements. All the documents from the database were cleaned with a set of normalization steps and the two feature extractions, and feature selection techniques used were BoW, TF-IDF and ${\mathrm{CHI}}^{2}$ respectively. The algorithms used for classification were Logist Regression (LR), Support Vector Machine (SVM), Multinomial Naive Bayes (MNB) and k-Nearest Neighbors (kNN). The novelty of our work is the data used to perform the experiment, the details of the steps used to reproduce the classification, and the comparison between BoW, TF-IDF and ${\mathrm{CHI}}^{2}$ for this repository not having been covered by other studies. This work will serve as a reference for the software engineering community and will help other researchers to understand the requirement classification process. We noticed that the use of TF-IDF followed by the use of LR had a better classification result to differentiate requirements, with an F-measure of 0.91 in binary classification (tying with SVM in that case), 0.74 in NF classification and 0.78 in general classification. As future work we intend to compare more algorithms and new forms to improve the precision of our models.

## 1. Introduction

Text classification is the attempt to organize text documents into categories based on properties and attributes belonging to each text. This task is widely seen as a supervised learning task that is defined as the identification of categories of new documents based on the probability suggested by a specified training corpus of already labelled (identified) documents [1]. Text classification is used in several domains, including spam identification and news categorization. The concept may seem simple and, with a small number of documents, it is possible to analyze each document manually and get an idea of the category in which the document belongs. Based on this knowledge, it is possible to group similar documents into categories or classes. It is a more challenging activity when the number of documents to be classified increases to several hundred thousand or millions. It is in this context that vectorization techniques and the supervised or unsupervised learning are useful. Document classification is a generic problem not limited to text alone but also can be extended for other items like music, images, video, and other media [2].

The task of Software Requirement Classification (SRC), consists of specifying the category to which a given Software Requirement (SR) belongs [3]. The categories of a requirement are defined in two types: Functional Requirements (FRs), which describe the services, behavior or functions that a system provides, and Non-Functional Requirements (NFRs), which include the attributes (such as quality, usability, security, privacy, etc.), or restrictions in the application to be developed or in the software development process [4].

Even with the software requirements being well known and well described, the automatic classification of requirements written in natural language into functional requirements and the subcategories of non-functional requirements is still a challenge. According to Abad et al. [5], this is particularly due to the fact that stakeholders, as well as requirements engineers, use different terminologies and sentence structures to describe the same kind of requirement. The high inconsistency in requirements elicitation makes automated classification more error-prone, so the problem is to find optimal ways to realize a good automated classification. Furthermore, such classification is needed because manually classification of software requirements is a time-consuming task especially on large projects with a huge number of requirements [6].

In this work, we will investigate how the classification of software requirements can be improved, analyzing which is the best text vectorization technique to be used, using the techniques known as Bag of Words (BoW), Term Frequency and Inverse Document Frequency (TF-IDF) [7], and Chi Squared (${\mathrm{CHI}}^{2}$) and what machine learning model [8] has the best performance in the task of classifying requirements. We choose to include Chi Squared because many research results show that feature selection can improve the performance of text classification [9], and because ${\mathrm{CHI}}^{2}$ is reported by many studies as one of the most effective algorithms [10]. There is no other article in the field of requirements classification that evaluates the same database comparing the techniques used in here. The results of this experiment can help developers that want to automatize the software requirements classification choose the techniques and algorithms that will most help them, and can help researches in this field as a reference or guideline for other studies.

The main contributions of this work were: Comparison between three important vectorization techniques, BoW, TF-IDF, and Chi Squared, and the comparison of the use of each one of these techniques combined with four supervised classification algorithms—Support Vector Machine (SVM), K-Nearest Neighbor (KNN), Multinomial Naive Bayes (MNB) and Logistic Regression (LR). Through these comparisons we demonstrate which is the best vectorization technique for software requirements (between BoW, TF-IDF and ${\mathrm{CHI}}^{2}$) and which is the best algorithm (between SVM, kNN, MNB and LR) to classify Software Requirements. This comparisons will serve as a reference for future works in the software requirements community. The differential of our work is the use of PROMISE_exp to perform the experiments, since there is no current studies performing this experiments with the same level of the details for this repository.

This paper is organized as follows: In the Section 2 are presented the concepts related to the techniques of pre-processing and feature extraction of text, machine learning algorithms, performance measures, and the related works. Section 3 presents the research method used in this study. The Section 4 describes the results that we found. The threats to validity of the study are discussed in Section 5. Finally, the conclusions and future work are presented in the Section 6.

## 2. Background and Related Works

In this Section we present some papers related to the analysis of requirements, including survey, classification and summarization of requirements.

Kurtanovic et al. [11], used the Support Vector Machines (SVM) algorithm to classify requirements into Functional Requirements (FR), Non-Functional Requirements (NFR), and the subcategories of non-functional requirements. The authors used the PROMISE repository, which has the characteristic of being unbalanced in terms of functional and non-functional requirements. To balance the database, user comments made on Amazon products were integrated into the main data set. The authors had a precision and recall up to 90% for automatically identifying FRs and NFRs and for the identification of specific NFRs.

A problem currently encountered in searches that need to use public databases containing software requirements is that there are a limited number of such databases available online. The most commonly used requirements database is the PROMISE repository [12]. However, this repository is unbalanced and has only 625 classified (labeled) requirements written in natural language. In order to expand the PROMISE public database for future research, Lima et al. [3] used Google search engines to search for Requirements Engineering (RE) documents, which subsequently went through a validation phase, where the consensus of some experts on the type of software requirement they determined was measured. The data obtained was used to expand the PROMISE database, forming the PROMISE_exp repository. The result of the expansion was evaluated, comparing classifications in both the original and the new base using SVM, Decision Tree, Multinomial Naive Bayes and kNN algorithms. The results showed that in most cases, PROMISE_exp has improved the rankings.

The leading App distribution platforms like Apple, App Store, Google Play, Windows, among others, have over 4 million applications [13]. Users can evaluate an App by giving a rating with a text feedback after they download and use the App. These feedbacks contain valuable information which may help developers to better understand user needs and complaints during software maintenance and evolution. Taking this into consideration, Lu et al. [10], automatically classified user ratings among four types of NFRs (reliability, usability, portability and performance), RF and others. The authors combined the use of four classification techniques: BoW, TF-IDF, CHI and AUR-BoW (proposed in the study), combined with three machine learning algorithms: Naive Bayes, J48, and Bagging. The authors concluded that the combination of the AUR-BoW technique with the Bagging algorithm presented the best performance, with an accuracy of 71.4%, recall of 72.3%, and F-measure of 71.8%.

Jindal et al. [14] performed an automated analysis of various software requirements specifications from the PROMISE repository and introduced binary classification into different types of security requirements categories using a single machine learning algorithm (the J48 decision tree). The authors used pre-processing techniques such as tokenization, stemming and stop word removal, and performed vectorization using TF-IDF. The result analysis indicated that all the four models have performed very well in predicting their respective type of security requirement.

### 2.1. Requirements Elicitation

A requirement it’s a need, functionality or characteristic of a system. Three are mainly three definitions for requirements [15]:

1. A condition or capability needed by a user to solve a problem or achieve an objective.
2. A condition or capability that must be met or possessed by a system or system component to satisfy a contract, standard, specification, or other formally imposed documents.
3. A documented representation of a condition or capability as in (1) or (2).

During the development process, requirements engineering must elicit the stakeholder’s requirements, document the requirements in a suitable manner, validate and verify the requirements, and manage the requirements over the course of the entire life cycle of the system [16]. Requirements elicitation is a critical activity that forms part of the requirements engineering process because it has to discover what the software must do through a solid understanding of the wishes and needs of the various stakeholders and to transform them into software requirements [17,18].

Pohl et al. [16] said that a core activity of requirements engineering is the elicitation of requirements for the system to be developed. The basis for requirements elicitation is the knowledge that has been gained during requirements engineering about the system context of the system to be developed, which comprises the requirements sources that are to be analyzed and queried [16]. To make a good documentation of the requirements, they are divided into categories, the main ones being: Functional Requirements and Non-Functional Requirements. These two types are explained in the Section 2.1.1 and Section 2.1.2.

#### 2.1.1. Functional Requirements

A definition from Pohl et al. [16] is that: A functional requirement is a requirement concerning a result of behavior that shall be provided by a function of the system. According to IEEE et al. [15], FRs are requirements that specifies a function that a system or system component must be able to perform. Fernandes et al. [19] said that a functional requirement describes a functionality to be made available to the users of the system, characterizing partially its behavior as an answer to the stimulus that it is subject to. This type of requirement should not mention any technological issue, that is, ideally functional requirements must be independent of design and implementation aspects [19].

#### 2.1.2. Non-Functional Requirements

Non-Functional requirements define desired qualities of the system to be developed and often influence the system architecture more than functional requirements do [16]. Another definition, according to Fernandes et al. [19], is that: A non-functional requirement corresponds to a set of restrictions imposed on the system to be developed, establishing, for instance, how attractive, useful, fast, or reliable it is. Anton et al. [20] defined NFRs as something that describes the non-behavioral aspects of a system, capturing the properties and constraints under which a system must operate and, according to Davis et al. [21], this category of requirement is a set of required overall attributes of the system, including portability, reliability, efficiency, human engineering, testability, understandability, and modifiability. NFRs can be divided into subcategories. Non-functional requirements sometimes are classified as product requirements, organizational or process-related requirements and external requirements [22,23]. The difference between non-functional and functional requirements, in Software Engineering, should hinge on “how” and “what” systems perform or offer as resources [22]. The repository PROMISE considers 11 subcategories for this type of requirement. The type and description of each subcategory is explained in Table 1 [11]:

### 2.2. Machine Learning in Software Engineering

Machine Learning is the science (and art) of programming computers so they can learn from data.Fundamentally, machine learning involves building mathematical models to help understand data [24]. Currently, machine learning has been used in several contexts, such as: Analyzing images of products on a production line to automatically classify them [25], detecting tumors in brain scans [26], automatically classifying news articles [27], detecting credit card fraud [28], creating a chatbot or a personal assistant [29], and others.

Recently the use of Machine Learning (ML) within software engineering has been studied for both management and software development [3]. To carry out these studies, data repositories originating from the software development process, such as forum discussions, maintenance history, user feedback and comments from users on social networks have become a rich source of data for ML use, combined with text analysis to extract useful information that can be used in the future. Iqbal et al. [12] have collected a great amount of research related to the link between software engineering and machine learning. The authors found that ML impacts the activities of elicitation, analysis, validation and requirements management.

In this work, we will use text normalization, feature extraction techniques and machine learning algorithms to create software requirements classification models, using the PROMISE_exp [3] database. Unlike previous work, we will investigate which feature extraction technique and which algorithm has the best accuracy to perform requirements classification using the PROMISE_exp database.

### 2.3. Text Normalization

Text normalization is defined as a process that consists of a series of steps that should be followed to wrangle, clean, and standardize textual data into a form that could be consumed by other Natural Language Processing (NLP) and analytics systems and applications as input [2]. One of these steps is tokenization, which consists of dividing a text into a list of tokens, and these tokens can be sentences or individual words, depending on the researcher’s choice. Often, the tokenization itself is also part of the normalization of the text. Besides tokenization, several other techniques are part of the normalization process, such as: Case conversion, spelling correction, removal of irrelevant words and other unnecessary terms, such as articles and pronouns, stemming and lemmatization.

In this work, the database containing the software requirements went through the normalization process where the documents in the corpus were tokenized, the texts were all converted to lower case, irrelevant words were removed, and the conjugated words in verb tenses were converted to their original forms using lemmatization.

### 2.4. Vectorization of Text

Machine learning algorithms operate on a numeric feature space, expecting input as a two-dimensional array where rows are instances and columns are features [7]. In order to perform machine learning on text, we need to transform our instances, documents, into vector representations such that we can apply numeric machine learning. The process of encoding documents in a numeric feature space is called feature extraction or more simply, vectorization and is an essential first step towards language aware analysis [7].

The Vector Space Model is a concept and model that is very useful in case we are dealing with textual data. According to Bengfort et al. [2], VSM is very popular in information retrieval and document ranking. VSM is an algebraic and mathematical model to represent texts as numerical vectors. Some of these models used in research involving text analysis are: Bag of Words [30], TF-IDF [31], and Averaged Word Vectors [32]. In this work, we use BoW, TF-IDF and ${\mathrm{CHI}}^{2}$. We only use this three techniques because of it’s simplicity and because the dataset used is small and context is domain specific.

#### 2.4.1. Bag of Words

The Bag of Words model is perhaps one of the simplest yet most powerful techniques to extract features from text documents [2]. The essence of this model is to convert text documents into vectors such that each document is converted into a vector that represents the frequency of all the distinct words that are present in the document vector space for that specific document [2]. In summary, using BoW, a requirement “*j*” is expressed by a vector $X_{j}$ = $\left(x_{1,j}\;\ldots \;x_{i,j}\;\ldots \;x_{n,j}\right)$, in which $x_{i,j}$ denotes the weight of feature “*i*” calculated by the frequency of term “*i*” in requirement “*j*” and “*n*” denotes the number of terms in the dictionary [10]. After that, the manually classified requirements which are expressed by vectors act as input of supervised machine learning algorithms, and consequently are used to train classifiers [10].

#### 2.4.2. Term Frequency—Inverse Document Frequency (TF-IDF)

TF-IDF is a model that combines two metrics: (1) The raw frequency value of a term in a particular document; and (2) the inverse of the document frequency for each term, which is computed by dividing the total number of documents in our corpus by the document frequency for each term and then applying logarithmic scaling on the result. The inverse of the document frequency can be represented mathematically by the following formula:

$$idf_{i}=\log\frac{total\_requirements}{total\_requirements\_with\_term\_i}$$

Combining the two metrics, the TF-IDF feature vector can be mathematically defined as:

$$TF-IDF\left(term_{i,j}\right)=tf_{i,j}\times idf_{i}$$

where tf represents the frequency of the term and idf the inverse of the frequency of the document, for term *i* and document *j*.

### 2.5. Feature Selection

The text vectoring process in some cases generates weak informative features for the classification process, which may not help, or harm, the performance of ML algorithms. To solve this problem feature selection can be used as a new step in the process of representing text documents to reduce the native space without sacrificing categorization accuracy. A definition from Geron et al. [8] says that it’s a preparation data process where we drop the attributes that provide no useful information for the task. Feature selection is necessary to make large problems computationally efficient-conserving computation, storage and network resources for the training phase and for every future use of the classifier [33]. In our work, feature selection follows the principle that words that exist in more classes contain less class information, and consequently are less important for requirement classification.

#### 2.5.1. Chi Squared

Chi Squared (${\mathrm{CHI}}^{2}$), also known as $X^{2}$, is the common statistical test that measures divergence from the distribution expected if one assumes the feature occurrence is actually independent of the class value [33]. It measures the lack of independence between a term *t* and a class *c*. Yang et al. [9] defines the measure by the formula:

$$X^{2}\left(t,c\right)=\frac{N\times {\left(AD-CB\right)}^{2}}{\left(A+C)\times (B+D)\times (A+B)\times (C+D\right)}$$

where *N* is the total number of documents, *A* is the number of times *t* and *c* co-occur, *B* is the number of times the *t* occurs without *c*, *C* is the number of times *c* occurs without *t* and *D* is the number of times neither *c* nor *t* occurs.

### 2.6. Machine Learning Algorithms

Machine Learning (ML) has four main categories, namely: (1) supervised learning; (2) unsupervised learning; (3) semi-supervised learning; and (4) reinforcement learning [8]. In supervised learning, the training set used to feed the algorithm includes the desired solutions, called labels. On the other hand, in unsupervised learning the training data are unlabeled and the algorithm must learn without prior assistance.

The semi-supervised algorithms use partially labeled bases, and in learning by reinforcement, the system can observe the environment, select and perform actions, and receive rewards in return. The learning system, called an agent in this context, can observe the environment, select and perform actions, and get rewards in return (or penalties) [8]. In this work, we use the supervised learning category.

The supervised learning algorithms used in this research were:

1. k-NN (k-Nearest Neighbor). is based on the principle that the instances within a dataset will generally exist in close proximity to other instances that have similar properties [34]. The algorithm classifies a new data by calculating the distance of this data with the already existing instances inside the data base, then it selects the closest *k* instances and calculates their average, for regression problems, or gets the mode.
2. SVM (Support Vector Machines). Support Vector Machines are a particularly powerful and flexible class of supervised algorithms for classification and regression [24]. SVM is a powerful and versatile machine learning model, capable of performing linear or non-linear classification, regression and even outliers detection. The algorithm makes the classification creating a linear hyperplane of maximum margin that separates two classes. This margin makes there are few possibilities of separating the data from the sample, and thus, there is little chance of misclassifying new instances.
3. Logistic Regression. Logistic regression is commonly used to estimate the probability of an instance belonging to a specific class (for example, what is the probability that this email is a spam?) [8]. Abdul et al. [30] defined Logistic Regression as a regression class in which an independent variable is used to forecast the dependent variable. It is called binary logistic regression when the dependent variable has two classifications. It is called multinomial logistic regression when the dependent variable has more than two classes. In this work, we use binary logistic regression to classify the requirements between Functional Requirements and Non-Functional Requirements, and multinomial logistic regression to classify the subclasses of Non-Functional Requirements.
4. Multinomial Naive Bayes. A generative model estimates the conditional probability of a class given input data. In particular, Naive Bayes assumes the input features are independent each other (conditional independence). Multinomial Naive Bayes is a specialized version of Naive Bayes mostly used for document and text classification [35].

### 2.7. Performance Measures

Evaluation metrics are primarily used to evaluate the performance of a classifier. The performance is verified through mathematical formulas that will compare the predictions obtained by a model with the actual values in the database.

Precision measures the percentage between the quantity of correctly classified samples in relation to the total of samples. Precision is calculated using the ratio of the total of correct classifications to the total of classifications performed. This calculation can also be seen as the ratio of the quantity of true positives *(TP)* to the quantity of positives *(TP+FP)*:

(1) $$\mathrm{Precision}=\frac{TP}{TP+FP}$$

A precision equals to 1 indicates a percentage of 100% of the classifier and that all the instances were classified correctly.

Recall is a proportion of positive instances that are detected correctly by the classifier. It is calculated through the number of times that a class was correctly predicted *(TP)*, divided by the number of times that the class appears in the test data *(FN)*.

(2) $$\mathrm{Recall}=\frac{TP}{TP+FN}$$

It is often convenient to combine precision and recall into a single metric called the F1 score (also known as F-measure), in particular if you need a simple way to compare two classifiers [8]. The F1 score is the harmonic mean of precision and recall. Whereas the regular mean treats all values equally, the harmonic mean gives much more weight to low values. As a result, the classifier will only get a high F1 score if both recall and precision are high [8].

(3) $$F1-\mathrm{score}=\frac{2TP}{2TP+FP+FN}$$

Accuracy represents the fraction of correctly classified observations. It is a more general measure because it calculates the number of right classifications on the whole. Is a good measure when the target classes in the data are (nearly) balanced.

(4) $$\mathrm{Accuracy}=\frac{TP+TN}{TP+FP+TN+FN}$$

In this work, we will compare the performance of four machine learning algorithms, that are SVM [36], kNN [37], MNB [35] and LR [38], combined with BoW [30] and TF-IDF [31], separately, to classify the FRs and NFRs (and its sub-classes) of PROMISE_exp [3].

## 3. Method

This work aims to answer the following research questions (RQ):

RQ.1. Which feature extraction technique works best (BoW vs. TF-IDF vs. ${\mathrm{CHI}}^{2}$), for classifying Software Requirements into Functional Requirements and Non-Functional Requirements, and the sub-classes of Non-Functional Requirements?

RQ.2. Which Machine Learning Algorithm provides the best performance for the requirements classification task?

To answer RQ.1, we performed the conversion of the requirements into numerical vectors, each of these vectors was combined with the classification algorithms, and then we looked at BoW, TF-IDF and Chi Squared to see which one was the best. To answer RQ.2 we used the same results as the classifications obtained to answer RQ.1, but instead of comparing BoW, TF-IDF and Chi Squared, we compared the results obtained by SVM, kNN, MNB and LR, through performance measures, as presented in Section 2.7.

**Figure 1:**
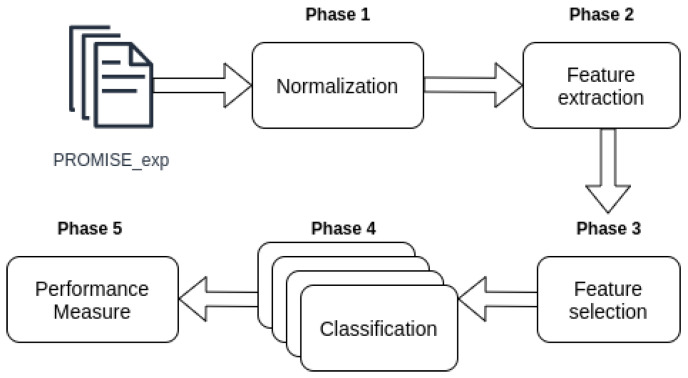
Phases of requirements classification pipeline.

We used four phases (steps) to perform the software requirements classification, as shown in Figure 1. The steps used were:

1. Normalization. This is the first step where the data is cleaned. All irrelevant words such as pronouns and articles are removed. Flexed verbs and nouns are converted to their root form (Phase 1 Figure 1).
2. Vectorization. Known also as feature extraction. At this stage the software requirements corpus (normalized) are converted into numerical vectors that best represent the information contained in those requirements. In this study, we use BoW and TF-IDF to perform this conversion (Phase 2 Figure 1).
3. Classification. In this step the vectors obtained in the Phase 2 are used to train and predict the classification models of the four algorithms used in this work: SVM, MNB, kNN and LR (Phase 4 Figure 1).
4. Evaluation. This is the final stage of classification where the results of the requirement’s labels predictions and the true labels of these requirements are used to calculate the performance measures, presented in Section 2.7.

**Figure 2:**
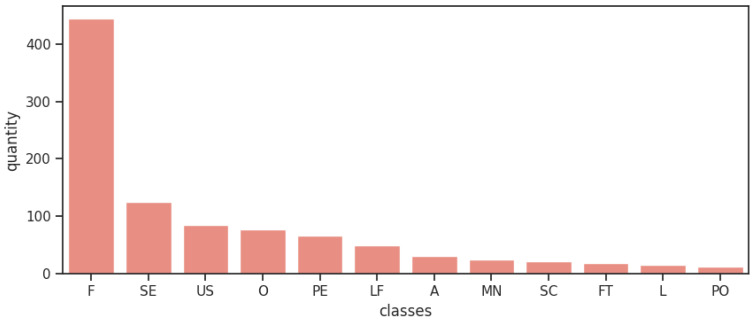
Distribution of requirement classes on dataset.

To perform the experiment, we used the programming language Python (https://www.python.org/) and the database PROMISE_exp [3]. This repository it’s a expanded version of the original PROMISE dataset, a public repository inspired by the UCI Machine Learning Repository, and created to encourage repeatable, verifiable, refutable, and/or improvable predictive models of software engineering [39]. The original repository consists of a pre-labeled set of 255 FRs and 370 NFRs, the last one being sub-classified into 11 different types of NFRs. The expanded version of Lima et al. [3] consists of 969 requirements and its composition is presented in Table 2. The expansion occurred with a focused web search for documents containing records of software requirements, where a analysis was made on such documents, and software requirements used in the expansion were identified and extracted. The entire process prioritized the compatibility and quality of the expansion and the expansion result was evaluated through the use of ML algorithms and its results were compared to the results of the original database when submitted to the same algorithms [3]. Figure 2 shows visually how unbalanced the classes are. The only case that classes are well distributed it’s when all sub-classes of NFRs are grouped in one unique class of NFRs when analysing the binary classification, such separation data results in 444 FRs and 525 NFRs.

All documents in the database have gone through a normalization process, this process is represented in Phase 1 “Normalization” of Figure 1. Table 3 shows the database requirements before the normalization process (cleaning of the text). We used a library called NLTK (https://www.nltk.org/) in the normalization process. This library is responsible for performing natural language processing. In this process, all words have been converted to lowercase letters, for example, Table 3 ID 1: “The system shall refresh the display every 60 s” has been changed to: “System shall refresh display every second”, as shown in ID 1 of Table 4. Then the words that had little or no significance were removed from the documents. These words are called stopwords and are usually the most common when we aggregate several documents. Words like “the”, “a” and so on are considered irrelevant words (Table 4).

The last step in the normalization process was to transform the conjugated words into their root form, for example, the word “users” was changed to “user” and “specified” was changed to “specify”. The code used for this step is showed in Listing 1. Table 4 shows the documents after the completion of the normalization step.

After performing the normalization, all documents went through the vectorization process, this phase is necessary so we can use the information extracted from the documents in our machine learning models, according to Phase 2 “Feature Extraction” of Figure 1. Thus, we use BoW and TF-IDF in the vectorization process.

The two forms of feature extraction (BoW and TF-IDF) were compared in the classification phase, where we can observe which of these techniques improved the performance of the algorithms used. In order to evaluate which words were considered the most influential, we counted the sum of the lines of each matrix resulted from vectorization. Table 5 presents the 10 words with highest score for each technique. It is possible to observe in the Table that the words are the same, but from the 3th position the order of importance changes from BoW to TF-IDF.

Phase 3 it’s a feature selection phase, where the features generated from feature extraction go through a process of filtering, to remove some features that are not so important according to some statistical approach, Chi Squared in our case. In BoW and TF-IDF vectorizations, the features filter was reached with an argument used in both feature extraction methods, max_df, that is used to ignore terms that have a document frequency strictly higher than the given threshold. Another argument was min_df that ignore terms that have a document frequency strictly lower than the given threshold, and the value of this argument was also achieved with.

In Chi Squared approach, we conducted a different filter for the features. We used the ${\mathrm{CHI}}^{2}$ technique (Section 2.5.1) for filtering the features obtained by TF-IDF. In our work, a function called SelectKBest was used. This function accepts two arguments, score_func, that is the score function chose (${\mathrm{CHI}}^{2}$) and k that is the *k* features with the best ${\mathrm{CHI}}^{2}$ that will be considered. To find the *k* value, we used the same hyperparameter optimization approach that finds the settings of BoW and TF-IDF.

The normalized and vectorized corpus was used for training and performance testing using four algorithms: kNN, SVM, MNB and LR (Phase 4 of Figure 1). These algorithms were used to classify software requirements into three different types of granularity: Functional Requirements and Non-Functional Requirements; sub-classes of Non-Functional Requirements; Functional Requirements and sub-classes of Non-Functional Requirements. In SVM, MNB and LR algorithms, a hyperparameter called class_weight was used, this hyperparameter uses the values of class labels to automatically adjust weights inversely proportional to class frequencies in the input data. All the hyperparameters, both from vectorization and classification algorithms, were chosen using a function called GridSearchCV. This function exhaustive search over specified parameter values for an estimator and chooses the best combination based in some scoring parameter, in our case was F-measure. In other words, this function test all the possible combinations between the parameters and returns the combination that achieved the best score. This winning combination is the combination that is used in the vectorization/classification process.

We calculated the performance of the four algorithms using cross-validation according to the Phase 5 “Performance Measure” of the classification process, presented in Figure 1. Cross-validation creates a sequence of fits where each subset of the data is used both as a training set and as a validation set [24], we choose this approach to deal with the unbalanced characteristic of the data. In this experiment, the corpus was divided into ten subsets (10-folds), of which 9 were used to train the algorithms—which corresponds to 90 percent of the base, and 1 was used to perform the tests, corresponding to 10 percent of the database [3]. From the cross-validation, we calculate the precision, recall and F-measure of the results. In the Listing 2 is the function created to perform the cross-validation, in this function we pass as parameters our Corpus (data), the classification algorithm (model), the object responsible for vectorizing the Corpus (vectorizer), and the number of folds (in our case are 10). The function therefore extract the features, trains and tests the data, printing at the end the performance measures obtained with the test.

The Scikit-learn (https://scikit-learn.org/stable/) tool was used to support the experiments. Scikit-learn is a Python module that integrates several machine learning algorithms to work with supervised and unsupervised problems. This tool was chosen because it contains the algorithms used in this study, as well as additional tools that implement BoW, TF-IDF and cross-validation. It is worth mentioning that this work differs from the work of [3] by including Logistic Regression in the list of classifiers to be analyzed, and removing Decision Trees from that list. In addition, our work performs a study on the differences of using BoW and TF-IDF in the classification of requirements [40].

## 4. Results

In this section, we present the results of the experiment and discuss its implications. We conducted three evaluations to determine the best combination of Machine Learning algorithm and feature extraction technique to classify requirements: (1) Effectiveness in binary classification of requirements; (2) effectiveness in multiclass classification of nonfunctional requirements; and (3) effectiveness in multiclass classification of requirements, including Non-Functional Requirements (NFRs) and Functional Requirements (FRs). In the context of these three experiments, we used the Machine Learning (ML) algorithms described in Section 2.6. The metrics used to evaluate the algorithms were: Precision Equation (1), Recall Equation (2) and F-measure Equation (3).

The performance evaluation of ML algorithms in binary classification of software requirements was conducted in order to determine the quality of the distinction between functional requirements and non-functional requirements. Table 6 presents the results obtained for the binary classification. It is possible to notice that SVM and LR had the best performance in the classification with its performance measures in the value of 0.91 using TF-IDF. SVM algorithm was also the algorithm that showed the least variation in its performances, with a performance greater or equal to 0.90 in all measures.

The MNB algorithm also had a great performance with TF-IDF. However, with a difference of 0.01, the F-measure of SVM and LR was higher. With this results, we can conclude that SVM and LR algorithms were better in binary classification of software requirements.

Table 7 presents the evaluation results of the ML algorithms used in the multiclass classification task of non-functional requirements. In this experiment, we evaluated the effectiveness of classifying the non-functional requirements in 11 different classes, according to labels originally defined in the PROMISE_exp database. In this classification, there was a significant decrease in the number of instances, since the database has 444 functional requirements, making the classification of the 11 classes of non-functional requirements have only 525 instances in its favor (969 of the corpus minus the 444 functional requirements).

Compared to the other granularities, the classifiers had a higher difficulty in differentiating functional requirements. The performance of the algorithms was worse compared to other granularities, mainly by the kNN algorithm combined with Bow, with a recall of 0.48. However, there was an improvement in the performance of the algorithms when using TF-IDF. According to the results, it is possible to identify that LR combined with TF-IDF was superior to SVM, MNB and kNN.

Table 8 presents an overview of the results of the experiment obtained by the three feature extraction techniques BoW, TF-IDF and Chi Squared combined with four machine learning algorithms SVM, MNB, kNN and LR, which show the weighted mean precision, recall and F-measure of the results of the classification into 12 different granularities (Functional Requirements and the 11 types of Non-Functional Requirements). In general, the precision, recall and F-measure of all combinations is greater or equal to 0.60.

LR algorithm achieved the best performance classification with 0.79 in recall measure. The most significant performance difference was in the kNN recall measure with an increase of 21.67% from BoW to TF-IDF. In the third case of granularity LR was the best algorithm.

In order to evaluate the classification obtained by the combination of TF-IDF and SVM, we analyzed the performance acquired by the algorithm for each of the labels in our database, as presented in Table 9. We can note that F was the requirement with the highest F-measure because of its large amount of observations.

It is also possible to observe a good classification of Performance requirements, with 0.82 in all measures, being the requirement with second largest F-measure even not being the class with the second higher number of observations. The worst classification was of the portability requirements, due to the smallest number of instances of those requirements in the PROMISE_exp. Another relevant observation is the performance in the classification of the security requirements, which despite of having a good amount of instances (125 instances), obtained a low precision in relation to its recall and F1-score. We believe that this has happened due to the classification becoming dependent on the appearance of keywords, such as “password” and “cookies”.

**Figure 3:**
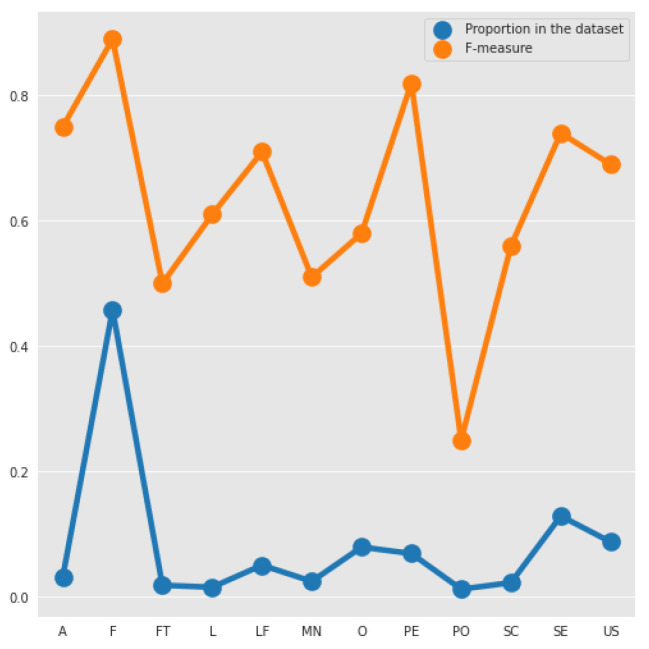
Comparison between the F-measure and proportion of each type in the dataset.

For a better visualization and comprehension, Figure 3 shows the relation between the proportion of each type of requirement and the F-measure obtained. We can notice that, there exists a relationship among these two measures, because the lines of them have a similar shape. We can see that for low proportion values, a slight increase or a big decrease in the number of examples, has a significant impact on the F-measure. Most of the F-measure’s of the requirements increases when the proportion increases, but unlike the other requirements the F-measure of Performance (PE) increases compared with Operability (O) when the number of examples decreases. Moreover, while the proportion differences of F to PE and A to SE are very significant, the F-measures of these tuples are very similar.

By analyzing the classifications for the three types of vectorization (BoW, TF-IDF and ${\mathrm{CHI}}^{2}$) to prepare the data for the 4 algorithms analyzed in this work (SVM, MNB, LR and kNN), we can state that TF-IDF as feature extraction, without Chi Squared, is a better vectorization technique than BoW and ${\mathrm{CHI}}^{2}$, which responds to RQ.1 defined in Section 3, by taking into account the number of times a given word appears in all documents, unlike BoW which only takes into account the frequency of that word in a document. In all cases, BoW proved to be better when combined with MNB, but with some of the measures having a difference of only 0.01. In the other cases the TF-IDF obtained an advantage. Moreover, LR proved to be the best algorithm when used with TF-IDF in all classification of requirements, tying with SVM in binary classification. In the classification with 11 granularities (non-functional requirements), the SVM proved to be better than LR, when using ${\mathrm{CHI}}^{2}$, but not when BoW and TF-IDF were used, but with equal performance measures or with a slight difference of 0.01 between them. In some cases, MNB was better than SVM, LR and kNN when combined with BoW, however, with the use of TF-IDF, LR was able to lead the classification, thus responding to the RQ.2 defined in Section 3.

## 5. Threats to Validity

In this section, we will discuss the limitations and threats to validity of this work, and how these threats were partially mitigated during the conduct of this research.

1. Database unbalance. Due to the unbalance, the classification obtained by the algorithms lost in performance, mainly in cases with 11 (Non-Functional Requirements) and 12 granularities (Functional Requirements and Non-Functional Requirements). Some non-functional requirements had a very small number of instances as it was the case of the Portability requirements, containing only 12 examples, which represents 1.24% of the PROMISE base. To get around the unbalanced, we use versions of the algorithms that take into account the bad distribution of classes and associate weights to those classes.
2. Reliability of the study. The reliability of the study is related to whether the research conducted will produce the same results as other researchers who replicate the study. In this work, reliability is related to the processes we adopt to perform the automatic classification of requirements. To mitigate this threat, we have detailed the processes adopted (Section 3). Thus, we believe that this threat has been partially mitigated.

## 6. Conclusions

In this work, we combined two text vectorization techniques with four machine learning algorithms to manually classify user requirements into two types (functional requirements and non-functional requirements), eleven types (non-functional requirements subclasses), and twelve types (functional requirements plus non-functional requirements subclasses). We evaluate the combination of these algorithms using the PROMISE_exp database, which is an expansion of the PROMISE database.

We conducted some experiments to compare the precision, recall and F-measure of the classification results across all combinations. We found that the combination of TF-IDF and LR has the best performance measures for binary classification, non-functional requirements classification and for requirements classifications in general, with an F-measure of 91% on the binary classification, 74% in 11 granularity classification and 78% on the 12 granularity classification.

Studies have shown that in an unbalanced data set, automatic classification performs worse when the size of requirements of some labels is smaller. Automatic Software Requirements classification can help developers document their projects more effectively, minimizing rework and making the software easier to use and understand. We hope that this study help developers to use this techniques to automatize software requirements categorization and help them to understand stakeholders needs. Furthermore, the classification performed can serve as a guideline and reference for other studies, helping specialists in the field to choose the classification algorithm that presents the best accuracy in the classification of NFR and FR requirements.

As future works, we aim to:

1. Increase the size of PROMISE_exp to use and explore others feature extraction techniques such as word2vec [41], AUR-BoW [10], Hierarchal BoW [42] and Online Unsupervised Multi-view Feature Selection [43].
2. Use advanced multi-view strategy to combine the different feature set in order to avoid redundant information.
3. Analyze the use of other supervised classification algorithms with the use of Neural Networks [44].
4. Compare the results of the supervised classification with those of an unsupervised classification.
5. Look for ways to mitigate the unbalance of the base, being able to improve the classification with little training data.

## Figures and Tables

**Listing 1:**
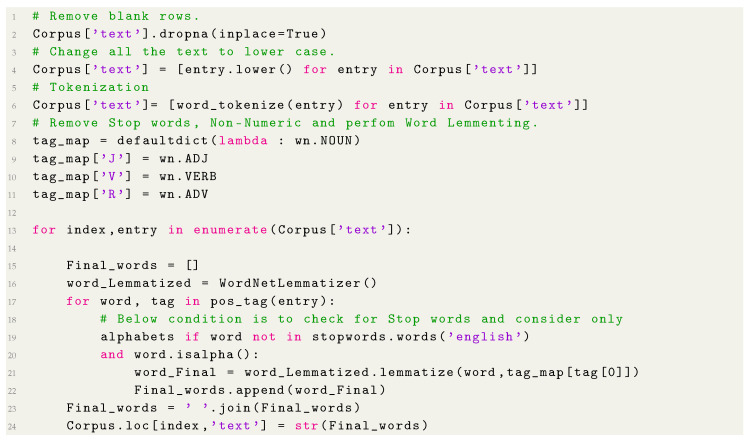
Corpus leaning.

**Listing 2:**
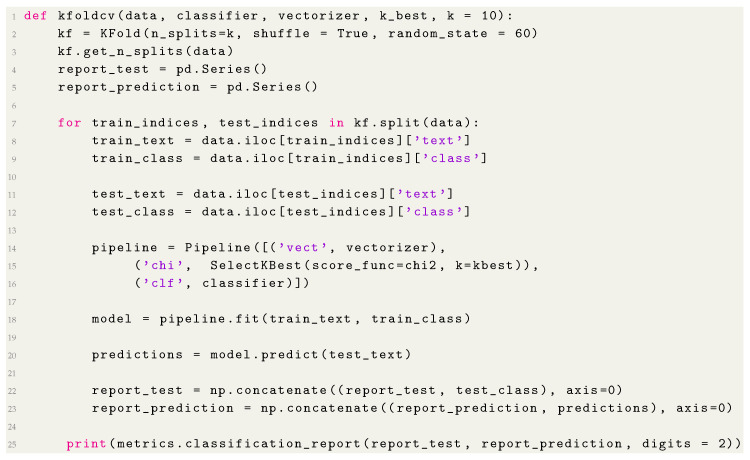
Classification with cross-validation.

**Table 1:** Descriptions of each type of Non-Functional Requirement (NFR) [11].

Type	Description
Availability (A)	Describes how likely the system is accessible for a user at a given point in time.
Fault Tolerance (FT)	Degree to which a system, product or component operates as intended despite the presence of hardware or software faults.
Legal & Licensing (L)	Certificates or licenses that the system must have.
Look & Feel (LF)	Describe the style of the product’s appearance.
Maintainability (MN)	Degree of effectiveness and efficiency with which a product or system can be modified by the intended maintainers.
Operability (O)	Degree to which a product or system has attributes that make it easy to operate and control.
Performance (PE)	Performance relative to the amount of resources used under stated conditions.
Portability (PO)	Degree of effectiveness and efficiency with which a system, product or component can be transferred from one hardware, software or other operational or usage environment to another.
Scalability (SC)	Degree to which a product or system can effectively and efficiently be adapted for different or evolving hardware, software or other operational or usage environments.
Security (SE)	Degree to which a product or system protects information and data, so that persons or other products or systems have the degree of data access appropriate to their types and levels of authorization.
Usability (US)	Degree to which a product or system can be used by specified users to achieve specified goals with effectiveness, efficiency, and satisfaction in a specified context of use.

**Table 2:** Number of requirements per label.

Type of Requirement	Number of Requirements
Functional Requirement (FR)	444
Availability (A)	31
Legal & Licensing (L)	15
Look & Feel (LF)	49
Maintainability (MN)	24
Operability (O)	77
Performance (PE)	67
Scalability (SC)	22
Security (SE)	125
Usability (US)	85
Fault Tolerance (FT)	18
Portability (PO)	12
Total	969

**Table 3:** Corpus before the text cleaning.

	Text	Class
1	The system shall refresh the display every 60 s.	PE
2	The application shall match the color of the schema set forth by Department of Homeland Security.	LF
3	If projected the data must be readable. On a 10 × 10 projection screen 90% of viewers must be able to read Event/Activity data from a viewing distance of 30.	US
4	The product shall be available during normal business hours. As long as the user has access to the client PC the system will be available 99% of the time during the first six months of operation.	A
5	If projected the data must be understandable. On a 10 × 10 projection screen 90% of viewers must be able to determine that Events or Activities are occurring in current time from a viewing distance of 100.	US
…	…	…
965	The system should be portable to various operating environments.	PO
966	Registered User must be able to maintain his/her session information for at least 60 min of inactive session before the system prompts him to log out of the system. The registered user must be provided with all the options of the E-store regardless of the time when he/she logs in.	F
967	The entire website must be user-friendly and easily navigable. The website must be provided with a site map for quick access to a particular link according to the requirement specification. The user must be able to find what he/she wants from the site without any difficulty. The website must adhere to branding schemes and the layout of the web pages must be uniform throughout.	US
968	The system shall support up to 10,000 simultaneous users against the central database at any given time and up to 5000 simultaneous users against the local servers at any one time. The performance of the website must be optimal increase of huge loads and hence appropriate load balancing must be done to achieve this. There can be any number of mirror servers readily available in case of huge loads without the user getting any delay.	PE
969	The website must provide highest degree of security to the registered users. All the transactions that are made must be secured. The sensitive information passed to and from the website must be secured. Identity theft and other security related issues must be solved. Unauthorized transmission of sensitive information of the user to third party websites for reference must be avoided. On the basis of user agreement the information must be processed. All the information about the registered user must be securely stored in the central database.	SE

**Table 4:** Corpus after the text cleaning.

	Text	Class
1	System shall refresh display every second.	PE
2	Application shall match color schema set forth department homeland security.	LF
3	Project data must readable projection screen viewer must able read event activity data view distance.	US
4	Product shall available normal business hour long user access client pc system available time first six month operation.	A
5	Project data must understandable projection screen viewer must able determine event activity occur current time view distance.	US
…	…	…
965	System portable various operate environment.	PO
966	Register user must able maintain session information least minute inactive session system prompt log system registered user must provide option regardless time log.	F
967	Entire website must easily navigable website must provide site map quick access particular link accord requirement specification user must able find want site without difficulty website must adhere brand scheme layout web page must uniform throughout.	US
968	System shall support simultaneous user central database give time simultaneous user local server one time performance website must optimal in case huge load hence appropriate load balancing must do achieve number mirror server readily available case huge load without user get delay.	PE
969	Website must provide high degree security registered user transaction make must secure sensitive information pass website must secure identity theft security relate issue must solve unauthorized transmission sensitive information user third party website reference must avoid basis user agreement information must process information registered user must securely store central database.	SE

**Table 5:** Top 10 most important features.

Position	BoW	TF-IDF
1°	must	must
2°	information	information
3°	user	sensitive
4°	website	website
5°	security	registered
6°	registered	security
7°	secure	secure
8°	sensitive	user
9°	high	third
10°	agreement	issue

**Table 6:** Results of Bag of Words (BoW), Term Frequency–Inverse Document Frequency (TF-IDF) and Chi Squared binary classification with Support Vector Machine (SVM), Multinomial Naive Bayes (MNB), k-Nearest Neighbors (kNN) and Logist Regression (LR).

	Binary Classification								
	**BoW**			**TF-IDF**			**${\mathrm{CHI}}^{2}$**		
	Precision	Recall	F1-score	Precision	Recall	F1-score	Precision	Recall	F1-score
SVM	0.90	0.90	0.90	0.91	0.91	0.91	0.90	0.90	0.90
MNB	0.91	0.91	0.91	0.91	0.91	0.90	0.89	0.89	0.89
kNN	0.82	0.82	0.82	0.87	0.87	0.87	0.84	0.84	0.84
LR	0.88	0.88	0.88	0.91	0.91	0.91	0.89	0.89	0.89

**Table 7:** Results of BoW, TF-IDF and Chi Squared classification with SVM, MNB, kNN and LR with 11 granularities (Non-Functional Requirements).

	Non-Functional Requirements Classification								
	**BoW**			**TF-IDF**			**${\mathrm{CHI}}^{2}$**		
	Precision	Recall	F1-score	Precision	Recall	F1-score	Precision	Recall	F1-score
SVM	0.68	0.67	0.66	0.73	0.73	0.72	0.72	0.71	0.71
MNB	0.71	0.73	0.72	0.71	0.71	0.71	0.69	0.70	0.68
kNN	0.56	0.48	0.49	0.66	0.66	0.66	0.62	0.63	0.62
LR	0.71	0.71	0.70	0.75	0.75	0.74	0.70	0.71	0.70

**Table 8:** Results of BoW, TF-IDF and Chi Squared classification with SVM, MNB, kNN and LR with 12 granularities (Functional Requirements and Non-Functional Requirements).

	Classification with 12 Granularities								
	**BoW**			**TF-IDF**			**${\mathrm{CHI}}^{2}$**		
	Precision	Recall	F1-score	Precision	Recall	F1-score	Precision	Recall	F1-score
SVM	0.73	0.73	0.72	0.78	0.78	0.78	0.77	0.77	0.76
MNB	0.77	0.77	0.77	0.76	0.77	0.76	0.74	0.74	0.73
kNN	0.63	0.60	0.60	0.72	0.73	0.72	0.67	0.69	0.68
LR	0.76	0.77	0.76	0.78	0.79	0.78	0.76	0.77	0.76

**Table 9:** Results of TF-IDF with SVM classifications (12 granularities—Functional Requirements and Non-Functional Requirements).

SVM Classification Using TF-IDF (12 Granularities)			
	Precision	Recall	F1-score
Availability (A)	0.77	0.74	0.75
Functional Requirement (F)	0.87	0.92	0.89
Fault Tolerance (FT)	0.70	0.39	0.50
Legal & Licensing (L)	0.88	0.47	0.61
Look & Feel (LF)	0.78	0.65	0.71
Maintainability (MN)	0.52	0.50	0.51
Operability (O)	0.64	0.53	0.58
Performance (PE)	0.82	0.82	0.82
Portability (PO)	0.50	0.17	0.25
Scalability (SC)	0.57	0.55	0.56
Security (SE)	0.71	0.77	0.74
Usability (US)	0.66	0.72	0.69
